# Laboratory Culture and Life Cycle of *Thelazia callipaeda* in Intermediate and Definitive Hosts

**DOI:** 10.3390/pathogens11091066

**Published:** 2022-09-19

**Authors:** Lingjun Wang, Di Li, Changzhu Yin, Hongri Tang, Bo Luo, Rong Yan, Yujuan Shen, Hui Liu

**Affiliations:** 1 Department of Parasitology, Zunyi Medical University, Zunyi 563000, China; 2 School of Life Sciences, Chongqing University, Chongqing 401331, China; 3 National Institute of Parasitic Diseases, Chinese Center for Disease Control and Prevention, Shanghai 200025, China

**Keywords:** *Thelazia callipaeda*, *Phortica okadai*, vector-borne zoonosis, animal model, intermediate host

## Abstract

Human thelaziasis caused by *Thelazia callipaeda* is being increasingly reported worldwide. Notably, an epidemic trend is observed in Southwest China. Whether *Phortica okadai* found in Southwest China can act as a vector of *T. callipaeda* and human-derived *T. callipaeda* animal infections has not been widely reported. Here, *P*. *okadai* was maintained in a laboratory and experimentally infected with first-stage larvae collected from adult *T. callipaeda* that were isolated from infected human subjects. Dead *P. okadai* were subjected to PCR assay and dissected every two days to detect *T. callipaeda*. Subsequently, live flies were used to infect a rabbit. The infection procedures were performed once a day (20 min) for two weeks. The results show that L1 collected from the adult *T. callipaeda* could successfully parasitize *P. okadai* captured in Zunyi, a city in Southwest China, and developed into L3, and a rabbit was successfully infected with *T. callipaeda* using *P. okadai* as the intermediate host. The present study demonstrates a human-derived *T. callipaeda* infection in rabbits, through *P. okadai*, under laboratory conditions for the first time. These results provide insights into the transmission cycle of *T. callipaeda* and constitute a foundation to develop an effective treatment protocol for *T. callipaeda* infection.

## 1. Introduction

Vector-borne *Thelazia callipaeda* (Spirurida, Thelaziidae) as a zoonotic nematode is of concern to public health because it can infect a wide range of host species, including dogs, wolves, and other mammals as well as humans [1]. *T. callipaeda* has been referred to as “oriental eyeworm” because it is found in humans and dogs from the Russian Federation and the Far East [2]. The adult *T. callipaeda* occurs under the eyelids, in the conjunctiva, and on the nictitating membrane of the eye. First-stage larvae (L1) are released in lachrymal secretions by female eyeworms after mating. After being consumed by secretophagous flies, L1 undergo two molts and transform into third-stage larvae (L3) in the vector testes, before migrating to the vector proboscis [3]. Flies feed on lachrymal secretions to transmit L3 to a new host. In approximately 35 days, L3 develop into adults [4]. It is believed that *T*. *callipaeda* larvae and adults play a role in the pathogenesis of ocular thelaziasis. The clinical manifestations of the infection include blepharospasm, discharge, and conjunctivitis [5]. *Phortica okadai* (Drosophilidae, Steganinae) is the intermediate host and vector for *T. callipaeda* in China, whereas *P. variegata* is a vector in Europe [6].

**Figure 1:**
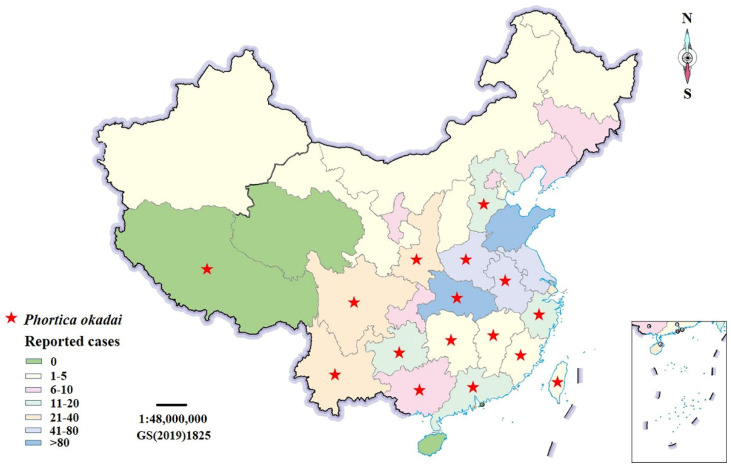
Map of human thelaziasis and *Phortica okadai* distribution in China. Note: The designations employed and presentation of the material on this map do not imply the expression of any opinion whatsoever on the part of Research Square concerning the legal status of any country, territory, city, or area or of its authorities, or concerning the delimitation of its frontiers or boundaries. This map has been provided by the authors.

Over the past two decades, various reports have highlighted *T. callipaeda* infections. Several human cases of *T*. *callipaeda* infection have been reported in southern, central, western, and eastern Europe [7,8]. There are also a few case reports in the United States; however, the vector has only been experimentally identified [6]. Beijing and Fujian were the first Chinese cities to report human thelaziasis [9,10]. As of 2021, China has documented 658 cases of *T. callipaeda* infection in 31 out of 34 provinces (except Tibet, Qinghai, and Hainan), autonomous regions, or municipalities (Figure 1) [11]. A significant increase in the number of reports in southwest China has been observed. However, unfortunately, in areas where *T. callipaeda* is endemic in China, the distribution of *P. okadai* has been confirmed in 16 provinces [12], and the distribution of *P. okadai* has not been found or has not been studied in the other 12 provinces (Figure 1).

The treatment protocols for thelaziasis are less complex than those for other parasitic infections such as trichinosis, and in most cases, worms are mechanically removed and macrocyclic lactones and mebendazole are administered [13]. Additionally, some clinicians and scientists may consider *T. callipaeda* of minor importance for the same reasons [10]. Clinical diagnosis of thelaziasis and its differentiation from allergic conjunctivitis, particularly when larval stages are present in the eyes, are challenging [14]. Concurrently, its ability to persist in an immunologically competent host could indicate that *T. callipaeda* has developed specific mechanisms to counter immune defenses [15]. However, the immune evasion mechanisms of *T. callipaeda* remain unclear. Establishing a reasonable and appropriate animal model is the premise for conducting research on *T. callipaeda*. Although many studies have reported the detection of *T. callipaeda* in definitive or intermediate hosts, few studies have established animal models, and literature reports lack specific morphological identification photos.

Hence, the aim of this study was (1) examine the method of establishing a *T. callipaeda*-infected animal model and describe the larval development of *T. callipaeda* in *P. okadai*, (2) verify whether *P. okadai* acts as a vector of *T. callipaeda* in Southwest China and (3) provide evidence for human-derived animal *T. callipaeda* infection. The present study constitutes a foundation to explore the immune escape mechanism of *T. callipaeda* and develop an effective treatment protocol for *T. callipaeda* infection.

## 2. Materials and Methods

### 2.1. T. callipaeda Collection and Identification

A man of 67 years from Guizhou province (26°57′27″ N, 107°21′38″ E) southwest of China without any history of travel in recent years visited a doctor in September 2021, at the Hospital of Zunyi Medical University. The patient had a history of foreign body sensations, conjunctival hyperemia, and increased eye secretions. Several nematode specimens were collected by an ophthalmologist using intraocular forceps, under anesthesia.

A morphological examination was performed under a microscope (Olympus BX43, Japan), followed by molecular identification. An extraction kit (M5 Hiper Universal DNA Mini Kit, MF033-plus, Zomanbio, China) was used to obtain genomic DNA from whole worms. A partial *cox*1 sequence of *T. callipaeda* (accession number: OP164161), approximately 200 bp in size, was amplified using a conserved primer (F: 5′-AGATGGCGTTTCCTCGTCT-3′, R: 5′-GCAAAGAACCAATACCCACAG-3′). Amplicons were amplified using a polymerase chain reaction (PCR) expansion kit (2xTaq Plus PCR MasterMix, TIANGEN Biotech Ltd., Beijing, China) and sequenced using an ABI3730XL DNA Analyzer (Applied Biosystems, Waltham, MA, USA). Genomic DNA (1 μL) was added to the PCR reaction mix (24 μL) with 9.5 μL ddH_2_O, 1 μL primers, and 12.5 μL Premix (2x). The PCR reaction system used the following cycling protocol: 94 °C for 3 min, 32 cycles of 94 °C for 30 s, 55 °C for 30 s, 72 °C for 60 s, 72 °C for 5 min, and 4 °C storage are followed. Using GenBank sequences of related nematodes with different haplotypes, the obtained sequence was genetically analyzed [16,17,18].

### 2.2. P. okadai Colonies

**Figure 2:**
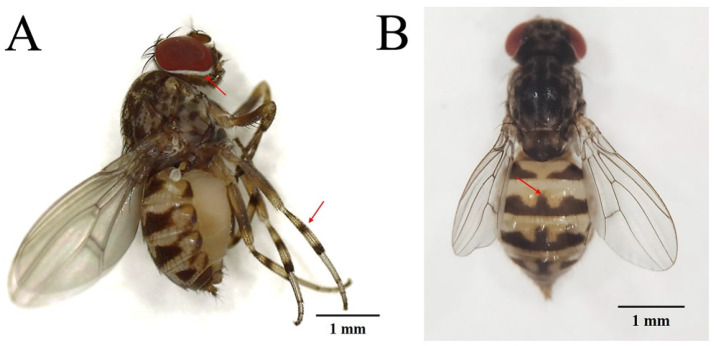
The typical morphological features(arrows) of *Phortica okadai*. (**A**) Abdomen of *Phortica okadai* showing three dark bands on the tibial band, a white ring around the eyes. (**B**) A “mountain” shaped black horizontal band on the dorsal side of the 3rd–5th abdominal segments.

*P. okadai* captured in Zunyi, a city in southwest China, was identified by Huang et al. in 2017 [19] with typical morphological features including three dark bands on the tibial band and a white ring around the eyes (Figure 2A) and a “mountain” shaped black horizontal band on the dorsal side of the 3rd–5th abdominal segments (Figure 2B) [20]. *P. okadai* was captured and then maintained in the laboratory of Zunyi Medical University within a well-sealed cage (22 × 22 × 27 cm) at 28 ± 2 °C, 75 ± 10% humidity, and 12/12 light/dark cycle. Water and pear, fermented for three days, were available ad libitum to the insects; water was changed daily.

### 2.3. Design and Analysis of P. okadai Infection Procedure

First-stage larvae were squeezed out of the mature female worms and placed on a slide with a drop of saline solution and observed under the light microscope (OLYMPUS DP260, Japan).

L1 were transferred to a concave slide with several drops of water after collection, and three-day-old, fermented pear juice (water-pear juice 1:1). Mature *P. okadai* (*n* = 100, F:M = 1:1), with food and water restricted 4 h prior, were used for experimental infection. The slide with L1 was placed in well-sealed cages (22 × 22 × 27 cm). Every 20 min, 1 mL of the medium was added to attract *P. okadai* to the feed. This process lasted for 2 h, after which the flies were normally fed (Supplementary Materials S1: Video S1: Chapter S3).

**Figure 3:**
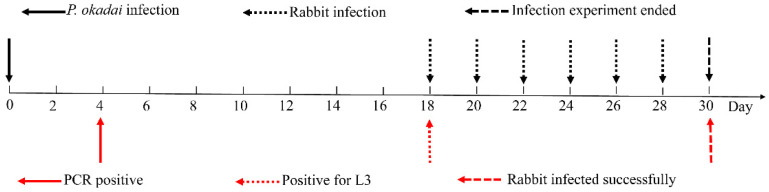
The infection and examination procedures.

Otranto et al. reported a procedure in which live and dead *P. okadai* were randomly collected every two days, examined via dissection, and subjected to molecular analysis with *cox*1 [21], as described above, until a positive infection was detected (Figure 3). Initially, the proboscis of the flies was stretched to detect positive L3, followed by dissection of the head, thorax, and abdomen with the purpose of detecting the presence or absence of other developmental stages of *T. callipaeda*.

### 2.4. Design and Analysis of Rabbit Infection Procedure

**Figure 4:**
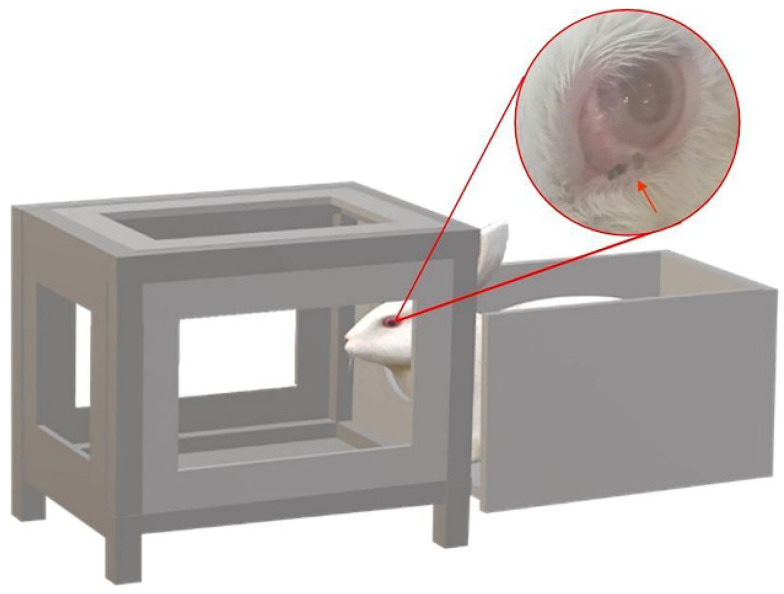
Device used during rabbit infection. A cage on the left and a fixed rabbit body on the right (the red arrow: *Phortica okadai* is feeding).

Approximately 4 h before experimentally infecting the female rabbit (6-week-old, 2.6 kg), the infected *P. okadai* were starved and dehydrated. We designed a device with a cage (22 × 22 × 27 cm) on the left and secured the rabbit’s body in the enclosure on the right (24 × 18 × 18 cm) (Figure 4). The infection procedures were performed once a day (20 min each time), with appropriate breaks in the middle, depending on the rabbit’s response (Supplementary Materials S1: Video S1: Chapter S5). The infection process lasted for two weeks, and the rabbit was observed daily for the presence of *T. callipaeda* worms. During this period, dead flies were collected and dissected every two days and examined.

## 3. Results

### 3.1. T. callipaeda Morphological Identification

**Figure 5:**
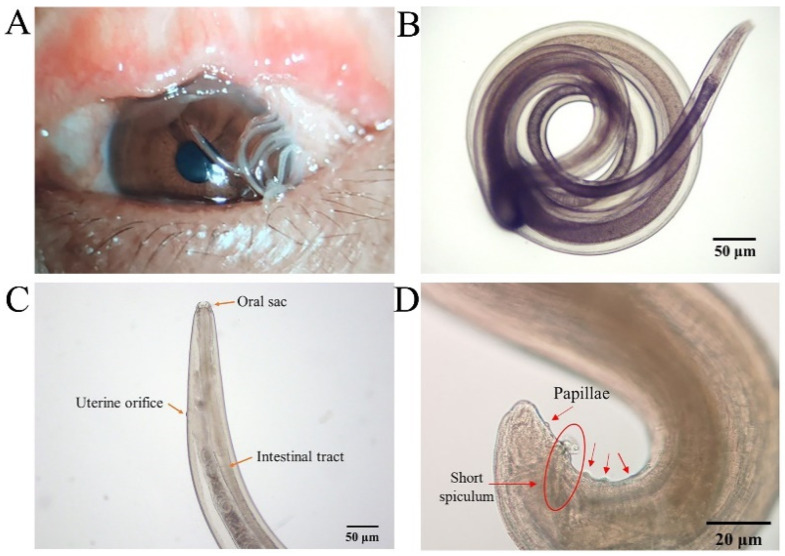
*Thelazia callipaeda* morphological identification. (**A**) The left eye of the patient with *Thelazia callipaeda* adult worms (*n* = 11, F:M = 7:4). (**B**) Adult male *Thelazia callipaeda* show a transparent spiral with a clearly visible internal digestive tract. (**C**) Anterior end of adult female *Thelazia callipaeda* showing oral sac, digestive tract, and coiled larvae in the uterus. (**D**) Posterior end of a male *Thelazia callipaeda* with non-protruding anal opening, post-anal papilla, and short copulatory spines.

Eleven worms were collected from a patient’s eyes (*n* = 11, F:M = 7:4) (Figure 5A, Supplementary Materials S1: Video S1: Chapter S1). The cuticle of their body wall showed a transparent spiral with a visible internal digestive tract under a light microscope (Figure 5B). The anterior end of the adult *T. callipaeda* has a polygonal oral sac with an elongated digestive tract and serrated cuticular striations. Coiled larvae in the twin-tube uterus were visible in the lower part of the head (Figure 5C). The tail of the female was straight and that of the male was ventrally curved with several pairs of papillae in front of the anus (Figure 5D). According to Rolbiecki [22,23], the worms were identified as *T. callipaeda* based on key morphological features. Four male and seven female *T. callipaeda* specimens were collected.

### 3.2. Molecular Analysis

**Figure 6:**
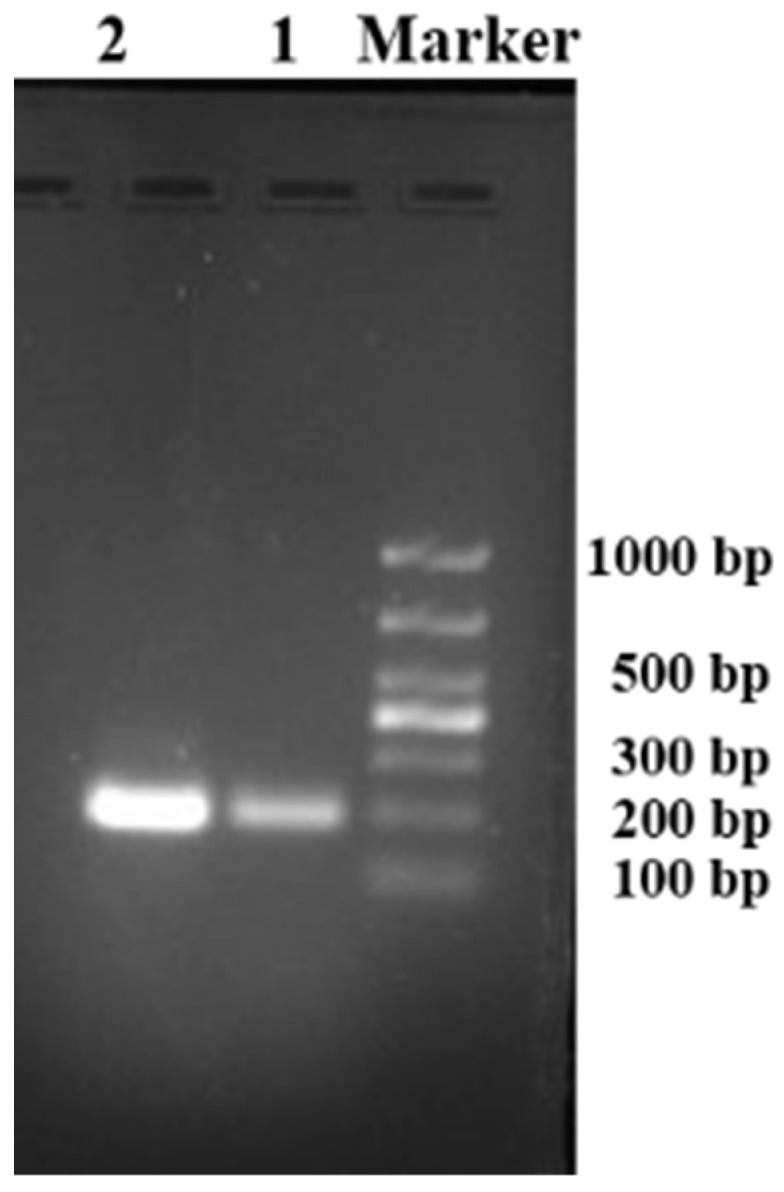
Polymerase chain reaction assay results of *Thelazia callipaeda cox1* (Marker: 1000 bp DNA Ladder, 1 and 2: infection of *Phortica okadai* DNA extract).

We cloned and amplified the *cox1* gene of the worm by PCR, which produced a 200 bp DNA fragment (Figure 6). Sequencing results showed that the characteristic *cox*1 gene was approximately 199 bp (accession number: OP164161). Since the gene sequence was only 199 bp, identification of the specific type of *T. callipaeda* was not possible; however, by analyzing the data (Supplementary Materials S2: Table S1), it could be inferred that the genotypes may belong to h3, h7, h15, h16, h18, h19, h20, and h21 (only h7 in Korea, others in China). The sequence similarity of this *cox*1 gene with that of European *T. callipaeda* haplotypes (h1 AM042549) was 97.4% and with that of Japanese *T. callipaeda* (h9–h12) was 97.4–99.4%.

### 3.3. Morphological Characteristics of L1

**Figure 7:**
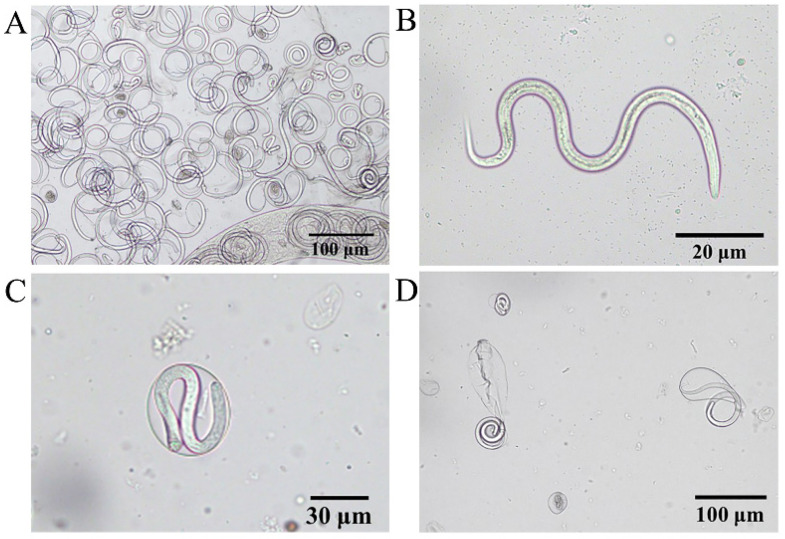
Morphological characteristics of L1. (**A**) Mid-section of adult female *Thelazia callipaeda* showing live larvae in the uterus and large numbers of newborn larvae. (**B**) *Thelazia callipaeda* newborn larvae showing a rounded head, a slender and pointed tail, and a complete digestive tract. (**C**) L1 coiled within intact follicular sacs. (**D**) L1 breaking out of the follicular sacs.

Due to artificial extrusion, numerous newborn larvae and larvae curled inside the capsule were visible under the microscope (Figure 7A). The L1 had a blunt and rounded head, a slender and pointed tail, a visible mouth capsule, and a complete digestive tract; the annulus formation had not yet begun, with a size of approximately (100–120) × 5 μm (Figure 7B). The larvae were curled in the oocyst, and the follicle size was approximately 40–50 μm (Figure 7C; Supplementary Materials S1: Video S1: Chapter S2); however, at this point, the larvae developed slower than the larvae follicle in a semi-ruptured state (Figure 7D) and were smaller in size.

### 3.4. Larval Development in P. okadai

**Figure 8:**
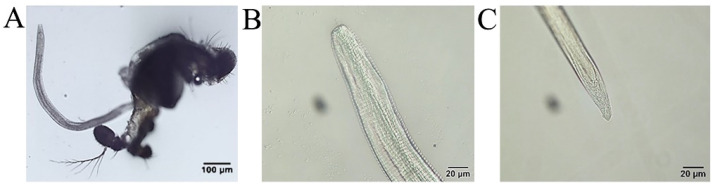
*Phortica okadai* dissection and *Thelazia callipaeda* observations. (**A**) L3 dissected from the mouthparts of *Phortica okadai*. (**B**) Anterior end of L3 of *Thelazia callipaeda* showing oral sac, digestive tract, and serrated folded body surface. (**C**) Posterior end of L3 of *Thelazia callipaeda* with a dorsal papilla and two lateral papillae.

On the fourth day following infection, the PCR results were positive (Figure 3), using the inspection procedure reported by Otranto et al. [21], which demonstrates the successful infection of *P*. *okadai* by *T*. *callipaeda*. One larva was micro-dissected from a female *P. okadai* on the 18th day; it had an elongated, transparent body, approximately 1294 μm × 30 μm in size, a serrated fold slightly under the head end and rounded (Figure 3, Figure 8A; Supplementary Materials S1: Video S1: Chapter S4), and blunt tail end with short copulatory spines that were faintly visible under high-power microscopy (Figure 8B). Ten (17.9%, *n* = 10, M: F = 8:2) out of 56 *P. okadai* were found with the L3, from the 18th to the 30th day (Supplementary Materials S2: Table S2).

### 3.5. Rabbit Infection Is Achievable

**Figure 9:**
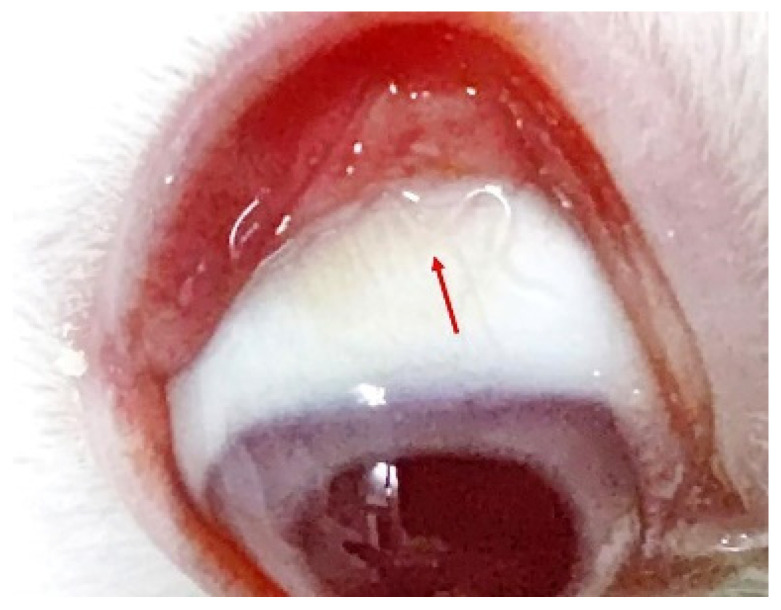
An adult *Thelazia callipaeda* (arrow) in the rabbit’s eye.

On the 12th day following the rabbit infection, a *T. callipaeda* larva was found in the right eye of the rabbit (Figure 9; Supplementary Materials S1: Video S1: Chapter S6). *T. callipaeda* was transparent, mostly hidden under the third eyelid, and swam freely in the conjunctival fornix. After 25 days, it reached adult size, and as it grew, no significant inflammation was observed in the rabbit’s eye.

## 4. Discussion

As far as we know, establishing a reasonable and appropriate animal model is the premise for conducting research on *T. callipaeda* and its life cycle is a necessity. This study established a rabbit model for *T. callipaeda* infection using L1 isolates from a human patient. The adults used in this study were not only identified by morphology but also by DNA sequence analysis of the *cox*1 gene. The molecular analysis results revealed 97.4% homology with the haplotypes (h1) found in Europe [24] and 100% homology with the worms in China and Korea [25]. Despite being a laboratory trial infection, unlike earlier cases of animal-derived human infections [26,27], this study is the first to report human-derived animal *T. callipaeda* infection. This study makes a significant contribution to the development of an animal model for *T. callipaeda* infection.

An important aspect of understanding thelaziosis associated with *T. callipaeda* is identifying the intermediate host; it includes both the insect species acting as the vector and the period when the larvae develop in the intermediate host. *T. callipaeda* was identified using a PCR assay performed on the fourth day of *P. okadai* infection, and flies were dissected on the 18th day (Figure 3 and Figure 8). These results are consistent with those of Otranto [21]. The infection rate of the male *P. okadai* was greater than that of females, which is also confirmed by Wang et al. [28]. Infected *P. okadai* mostly contained one larva, but some were also found to contain five larvae.

Temperature affects the rate of *P. okadai* infection and the development of L1 to L3. Wang et al. [28] have shown that, at 23.4–29.7 °C, the infection rate of *Drosophila* was the highest (53.49%), and the developmental cycle of L1 to L3 was the shortest (14 days). Ten (17.9%, *n* = 10, M: F = 8:2) of the 56 *P. okadai* were found with L3, from the 18th to the 30th day of our study. Different stages of larval development were found. This discrepancy could be explained by the fact that in Wang’s research, in our study, flies were dissected only on specific dates to retrieve infective larvae, rather than every day.

Notably, the number of *P. okadai* insects that died after being infected for 20 days gradually increased. Almost no L3 were found in the dead insects after 25 days and dissected flies revealed that the L3 were so large that it was difficult for them to burst out from the proboscis of *P. okadai*. Therefore, we estimated that the large body size of L3 interfered with feeding, resulting in the death of *P. okadai*. This may also explain why only one *T. callipaeda* was found in the natural environment simulated in this experiment. Previous research has shown that parasites are difficult to spot when they are in the larval stages or when they are less in number [21]. Therefore, the rabbit was diagnosed by directly observing the worms in both eyes (Supplementary Materials S1: Video S1: Chapter 6). This work provides evidence that *P. okadai*, collected from the Guizhou Province and experimentally infected with L1, may act as a possible vector for *T. callipaeda* in Southwest China. Concurrently, the current protocol used in the present study to rear *P. okadai* under laboratory conditions is a useful tool for morphological and behavioral investigations, such as “lachryphagy”.

In the past three years, the COVID-19 pandemic has taught us the relevance of integrative medicine (also known as One Health) [29,30]. Thelaziasis caused by *T. callipaeda*, as a vector-borne zoonotic parasitic disease, has also received growing interest. However, with urbanization, the entry of wildlife into urban areas, animal movements between regions, countries, and continents, and human leisure activities, diverse wildlife-domestic animal-human interfaces have been created. Therefore, the prevention and control of *T. callipaeda* infections are proving to be challenging [31].

China covers almost all ecosystems in the world, and many native or non-native species have found suitable habitats [32]. In addition to rapid economic development, the implementation of the “Silk Road Economic Belt” strategy has also greatly increased the risk of *T. callipaeda* transmission. To date, except for macrocyclic lactones such as milbemycin oxime and moxidectin with imidacloprid, no other effective drugs to prevent the infection of *T. callipaeda* in endemic areas have been found [33,34,35]. Therefore, the development of novel drugs and treatment strategies is necessary. The rabbit model in the present study provides an important platform for anti-*T. callipaeda* drug research in a preclinical setting and provides insights for future studies of immune evasion mechanisms of *T. callipaeda*.

Collectively, our findings suggest that *T. callipaeda* can be transmitted from humans to animals and that the L1 collected from female *T. callipaeda* is also infectious to *P. okadai* captured in Southwest China. The methods used make extensive contributions to establishing a suitable animal model for *T. callipaeda* infection.

## 5. Conclusions

In this study, we successfully established a rabbit model of *T. callipaeda* using *P. okadai* as a vector under laboratory conditions. The present study is the first to provide photographic evidence for the cross-transmission of *T. callipaeda* between humans and animals. It also suggests a likely expanding trend of this zoonotic nematode in Southwest China. The methods used in this study provide a reference for establishing other animal models of *T. callipaeda*. The establishment of a rabbit model also provides an important platform to explore the immune escape mechanism of *T. callipaeda* and a tool to develop novel drugs and treatment strategies for thelaziasis.

## Data Availability

All supporting data and protocols have been provided within the article or through Supplementary Data Files.

## Supplementary Materials

The following supporting information can be downloaded at: https://www.mdpi.com/article/10.3390/pathogens11091066/s1, Table S1: *cox*1 (199 bp) the h1–h21 sequence identity matrix; Table S2: Number and percentage of *P. okadai* (female-F/male-M, Live-L/Death-D) insects experimentally infected and found positive of *T. callipaeda* at dissection on different dates after infection.; Video S1: Process of *T. callipaeda* infection in rabbits.
