# Supplementary material for: Laboratory Culture and Life Cycle of Thelazia callipaeda in Intermediate and Definitive Hosts
Source: Pathogens. 2022 Sep 19;11(9):1066. doi: 10.3390/pathogens11091066 (PMC9504212; doi:10.3390/pathogens11091066)
Supplement: Supplementary file 1 [file pathogens-11-01066-s001.zip › Supplementary Materials_S2.pdf]

# Supplementary Material

**Table S1.** *cox*1 (199bp) with h1-h21 sequence identity matrix.

| ID | COX1 | h1 | h2 | h3 | h4 | h5 | h6 | h7 | h8 | h9 | h10 | h11 | h12 | h13 | h14 | h15 | h16 | h17 | h18 | h19 | h20 | h21 |
|---|---|---|---|---|---|---|---|---|---|---|---|---|---|---|---|---|---|---|---|---|---|---|
| COX1 | | | | | | | | | | | | | | | | | | | | | | |
| AM042549.1 h1 (Europe) | 0.974 | | | | | | | | | | | | | | | | | | | | | |
| AM042550.1 h2 (China) | 0.989 | 0.964 | | | | | | | | | | | | | | | | | | | | |
| AM042551.1 h3 (China) | 1 | 0.974 | 0.989 | | | | | | | | | | | | | | | | | | | |
| AM042552.1 h4 (China) | 0.984 | 0.979 | 0.974 | 0.984 | | | | | | | | | | | | | | | | | | |
| AM042553.1 h5 (korea) | 0.994 | 0.969 | 0.984 | 0.994 | 0.979 | | | | | | | | | | | | | | | | | |
| AM042554.1 h6 (korea) | 0.989 | 0.964 | 0.979 | 0.989 | 0.974 | 0.984 | | | | | | | | | | | | | | | | |
| AM042555.1 h7 (korea) | 1 | 0.974 | 0.989 | 1 | 0.984 | 0.994 | 0.989 | | | | | | | | | | | | | | | |
| AM042556.1 h8 (korea) | 0.989 | 0.964 | 0.979 | 0.989 | 0.974 | 0.984 | 0.989 | 0.989 | | | | | | | | | | | | | | |
| AB852543.1 h9 (korea) | 0.994 | 0.969 | 0.984 | 0.994 | 0.979 | 1 | 0.984 | 0.994 | 0.984 | | | | | | | | | | | | | |
| AB852550.1 h10 (korea) | 0.994 | 0.969 | 0.984 | 0.994 | 0.979 | 1 | 0.984 | 0.994 | 0.984 | 1 | | | | | | | | | | | | |
| AB852544.1 h11 (korea) | 0.974 | 0.949 | 0.964 | 0.974 | 0.959 | 0.969 | 0.964 | 0.974 | 0.964 | 0.969 | 0.969 | | | | | | | | | | | |
| AB852545.1 h12 (korea) | 0.974 | 0.949 | 0.964 | 0.974 | 0.959 | 0.969 | 0.964 | 0.974 | 0.964 | 0.969 | 0.969 | 1 | | | | | | | | | | |
| MF795664.1 h13 (China) | 0.989 | 0.974 | 0.989 | 0.989 | 0.984 | 0.984 | 0.979 | 0.989 | 0.979 | 0.984 | 0.984 | 0.964 | 0.964 | | | | | | | | | |
| MF795670.1 h14 (China) | 0.994 | 0.969 | 0.984 | 0.994 | 0.979 | 0.989 | 0.994 | 0.994 | 0.994 | 0.989 | 0.989 | 0.969 | 0.969 | 0.984 | | | | | | | | |

| | | | | | | | | | | | | | | | | | | | | | |
|---|---|---|---|---|---|---|---|---|---|---|---|---|---|---|---|---|---|---|---|---|---|
| MF795691.1 h15 (China) | 1 | 0.974 | 0.989 | 1 | 0.984 | 0.994 | 0.989 | 1 | 0.989 | 0.994 | 0.994 | 0.974 | 0.974 | 0.989 | 0.994 | | | | | | |
| MF795675.1 h16 (China) | 1 | 0.974 | 0.989 | 1 | 0.984 | 0.994 | 0.989 | 1 | 0.989 | 0.994 | 0.994 | 0.974 | 0.974 | 0.989 | 0.994 | 1 | | | | | |
| MF795678.1 h17 (China) | 0.994 | 0.979 | 0.984 | 0.994 | 0.989 | 0.989 | 0.984 | 0.994 | 0.984 | 0.989 | 0.989 | 0.969 | 0.969 | 0.994 | 0.989 | 0.994 | 0.994 | | | | |
| MF795680.1 h18 (China) | 1 | 0.974 | 0.989 | 1 | 0.984 | 0.994 | 0.989 | 1 | 0.989 | 0.994 | 0.994 | 0.974 | 0.974 | 0.989 | 0.994 | 1 | 1 | 0.994 | | | |
| MF795686.1 h19 (China) | 1 | 0.974 | 0.989 | 1 | 0.984 | 0.994 | 0.989 | 1 | 0.989 | 0.994 | 0.994 | 0.974 | 0.974 | 0.989 | 0.994 | 1 | 1 | 0.994 | 1 | | |
| MF795689.1 h20 (China) | 1 | 0.974 | 0.989 | 1 | 0.984 | 0.994 | 0.989 | 1 | 0.989 | 0.994 | 0.994 | 0.974 | 0.974 | 0.989 | 0.994 | 1 | 1 | 0.994 | 1 | 1 | |
| MF795694.1 h21 (China) | 1 | 0.974 | 0.989 | 1 | 0.984 | 0.994 | 0.989 | 1 | 0.989 | 0.994 | 0.994 | 0.974 | 0.974 | 0.989 | 0.994 | 1 | 1 | 0.994 | 1 | 1 | 1 |

**Table S2.** Number and percentage of *Phortica okadai* (female-F/male-M, Live-L/Death-D) that were experimentally infected and found positive of *Thelazia callipaeda* at dissection on different days post-infection

| Days post-infection | Number of flies | Flies collected | | | | Dissected flies positive for L3 | | | |
|---|---|---|---|---|---|---|---|---|---|
| | | Live | | Death | | Live | | Death | |
| | | Male | Female | Male | Female | Male | Female | Male | Female |
| 18 | 6 | 2 | 1 | 2 | 1 | 0 | 0 | 0 | 1 |
| 20 | 6 | 1 | 1 | 2 | 2 | 0 | 0 | 2 | 0 |
| 22 | 8 | 2 | 1 | 2 | 3 | 0 | 0 | 1 | 1 |
| 24 | 6 | 1 | 2 | 2 | 1 | 0 | 0 | 1 | 0 |
| 26 | 8 | 1 | 2 | 3 | 2 | 0 | 0 | 2 | 0 |
| 28 | 8 | 2 | 2 | 2 | 2 | 0 | 0 | 2 | 0 |
| 30 | 14 | 5 | 6 | 2 | 1 | 0 | 0 | 1 | 0 |
| Total | 56 | 14 | 15 | 15 | 12 | 0 | 0 | 8 | 2 |
| Infection rate | 17.9% | | | | | | | | |
